# Numerical Investigation of Non-Newtonian Fluid Rheology in a T-Shaped Microfluidics Channel Integrated with Complex Micropillar Structures Under Acoustic, Electric, and Magnetic Fields

**DOI:** 10.3390/mi16121390

**Published:** 2025-12-08

**Authors:** Muhammad Waqas, Arvydas Palevicius, Cengizhan Omer Senol, Giedrius Janusas

**Affiliations:** Faculty of Mechanical Engineering and Design, Kaunas University of Technology, 51424 Kaunas, Lithuania; cengizhan.senol@ktu.edu (C.O.S.); giedrius.janusas@ktu.lt (G.J.)

**Keywords:** microfluidics, non-Newtonian fluids, rheological behavior, shear-thinning, acoustic field, electric field, magnetic field

## Abstract

Microfluidics is considered a revolutionary interdisciplinary technology with substantial interest in various biomedical applications. Many non-Newtonian fluids often used in microfluidics systems are notably influenced by the external active fields, such as acoustic, electric, and magnetic fields, leading to changes in rheological behavior. In this study, a numerical investigation is carried out to explore the rheological behavior of non-Newtonian fluids in a T-shaped microfluidics channel integrated with complex micropillar structures under the influence of acoustic, electric, and magnetic fields. For this purpose, COMSOL Multiphysics with laminar flow, pressure acoustic, electric current, and magnetic field physics is used to examine rheological characteristics of non-Newtonian fluids. Three polymer solutions, such as 2000 ppm xanthan gum (XG), 1000 ppm polyethylene oxide (PEO), and 1500 ppm polyacrylamide (PAM), are used as a non-Newtonian fluids with the Carreau–Yasuda fluid model to characterize the shear-thinning behavior. Moreover, numerical simulations are carried out with different input parameters, such as Reynolds numbers (0.1, 1, 10, and 50), acoustic pressure (5 Mpa, 6.5 Mpa, and 8 Mpa), electric voltage (200 V, 250 V, and 300 V), and magnetic flux (0.5 T, 0.7 T, and 0.9 T). The findings reveal that the incorporation of active fields and micropillar structures noticeably impacts fluid rheology. The acoustic field induces higher shear-thinning behavior, decreasing dynamic viscosity from 0.51 Pa·s to 0.34 Pa·s. Similarly, the electric field induces higher shear rates, reducing dynamic viscosities from 0.63 Pa·s to 0.42 Pa·s, while the magnetic field drops the dynamic viscosity from 0.44 Pa·s to 0.29 Pa·s. Additionally, as the Reynolds number increases, the shear rate also rises in the case of electric and magnetic fields, leading to more chaotic flow, while the acoustic field advances more smooth flow patterns and uniform fluid motion within the microchannel. Moreover, a proposed experimental framework is designed to study non-Newtonian fluid mixing in a T-shaped microfluidics channel under external active fields. Initially, the microchannel was fabricated using a high-resolution SLA printer with clear photopolymer resin material. Post-processing involved analyzing particle distribution, mixing quality, fluid rheology, and particle aggregation. Overall, the findings emphasize the significance of considering the fluid rheology in designing and optimizing microfluidics systems under active fields, especially when dealing with complex fluids with non-Newtonian characteristics.

## 1. Introduction

In recent years, microfluidics has emerged as a dynamic and adaptable technology in a combination of physics, engineering, and chemistry, providing a powerful platform for precise fluid manipulation and control at the microscale [1,2]. Microfluidics technology has captivated the attention of researchers because of its wide range of applications, including biological diagnostics, drug delivery, and chemical analysis. One of the primary advantages of microfluidics technology is its ability to control tiny volumes of samples precisely and efficiently while performing complicated operations such as mixing, separation, detection, and actuation in a highly compact system [3]. In addition, microfluidics is a hastily growing technology because of its unique features, such as a high surface-to-volume ratio and better heat and mass transfer. These features support innovative ideas in numerous scientific and commercial domains while also offering high efficiency and low cost in different biomedical applications [4]. The advancement of microfluidics technology has made it possible to integrate multiple physical effects such as acoustic fields, electric fields, and magnetic fields into a single device, enabling researchers to study and exploit interactions that are very difficult to achieve in traditional large-scale systems [5,6]. With the advancement of this technology, it has become obvious that the behavior of fluids while flowing though the microchannels does not always follow the same predictable patterns that are observed at the macroscale. In particular, the rheological behavior of flowing fluids, especially when they are non-Newtonian in nature, can have a significant influence on how they move through the complex microchannel geometries. This makes it critical to thoroughly explore and comprehend the fluid dynamics of polymer solutions and other complex fluids employed in microfluidics systems [7].

Many types of fluids exhibit non-Newtonian behavior and are used in microfluidic systems for a wide range of applications. Non-Newtonian fluids are particularly interesting in terms of microfluidics because they exhibit a non-linear relationship between shear stress and shear rate compared to Newtonian fluids [8]. Moreover, the rheological behavior of non-Newtonian fluids becomes more challenging in complex microfluidic geometries compared to planar geometries. Many polymer solutions have shear-thinning and shear-thickening or viscoelastic characteristics, where their viscosity mainly depends on the flow behavior. These characteristics can strongly affect different microfluidics operations and change their flow rheology, which influences the overall microfluidics performance [9]. For example, in a microchannel designed for mixing operation with complex shape geometries, the performance may enhance or decrease depending on how the viscosity of the fluid varies with the shear rate. Because of these inimitable properties, non-Newtonian fluid polymer solutions are frequently used in many microfluidics systems for certain applications. At the same time, their complex flow rheology introduces new challenges in predicting and controlling fluid behavior in complex-shape microchannels, which are common in microfluidics designs.

Furthermore, studies show that many microfluidics systems are integrated with active and passive fields to carry out their operations in more efficient manners [10,11,12,13]. In active microfluidics systems, external effects such as acoustic [14], electric [15], magnetic [16], pressure [17], and thermal [18] effects are used to perform their operations, leading to enhanced efficiency. These effects create disturbances and enhance fluid interactions, resulting in increasing the shear rate, which greatly influences the flow rheology within the microchannels. Although integrating active fields offers many advantages such as rapid and controllable operations, at the same time, they also have many downsides such as using higher energy, especially during long operation, which makes experiments more complex and costly [19]. Moreover, microfluidics systems integrated with active fields require complicated laboratory setup and control systems, such as acoustic electrodes, electric fields, and magnetic controllers, etc. Ankit Kumar et al. [20] conducted a numerical investigation employing surface acoustic waves in order to improve the performance of microchannel flow for both Newtonian and non-Newtonian fluids. For this purpose, a three-dimensional computational model was developed to simulate the fluid flow within a rectangular cavity composed of PDMS bonded on a lithium niobate substrate. The findings revealed that surface acoustic waves enhanced the acoustic streaming, resulting in higher mixing and flow performance within a microchannel. Yanwen Gong et al. [12] used a contraction–expansion microchannel to conduct a numerical investigation to examine mixing performance, employing AC electroosmotic fields. The results highlighted the influence of flow velocities, frequency, and voltage on the vortex generation as well as mixing efficiency. Sara I. Abdelsis [21] investigated a peristaltic motion of electrically conducting Jaffrey fluid employed by an acoustic field in an enclosed parallel-plane microchannel integrated with a porous medium. It was determined that the oscillations declined quickly as the fluid transitioned from a hydrodynamic to a hydromagnetic fluid, and the influence of the delay time was also weakened. Muhammad Waqas et al. [3] proposed a novel passive-type micromixer integrated with a screw element in order to enhance the fluid mixing. The findings highlighted an improved mixing efficiency of up to 98% with the lowest pressure drop.

Alternatively, passive microfluidics systems do not need any external effects or energy sources but work primarily by controlling the flow through specific channel designs and features [22]. These designs typically disturb the flow streamlines within the microchannel, which increases the shear rate and alters the rheological behavior. Although passive microfluidics systems are energy-efficient and cost-effective, depending on flow manipulation through specific channel designs, they offer less efficiency and limited flow control compared to active systems. Honglin Lv et al. [23] conducted an optimization study of micromixers with Cantor fractal baffle features using a single-objective optimization approach. They used the Latin hypercube sampling (LHS) technique as an experimental design for parametric study. In addition, they also used response surface functions in order to approximate the mixing performance. Amar Kouadri et al. [24] investigated a passive kind of micromixer with different design configurations with the same hydraulic diameters and equivalent unfolded length. For this purpose, they performed numerical simulations at low Reynolds numbers using CFD Fluent code. They used non-Newtonian shear-thinning fluids (CMC solutions) employed by power-law fluid models with different power index ranges (0.73 to 1). The findings highlighted the Two-Layer Crossing Channels Micromixer (TLCCM) as the best micromixer design with a 96% mixing efficiency. Shuai Yuan et al. [25] conducted a numerical analysis of a novel planar microfluidics micromixer with staggered Z-shaped baffles in order to achieve rapid mixing and optimize the pressure drop. Harshad Sanjay Gaikwad et al. [26] investigated the influence of viscoelastic fluid on the improvement in electrochemomechanical energy conversion performance in polyelectrolyte layer grafter narrow fluidic channels. They revealed that the shear-thinning behavior of the viscoelastic fluid increase the flow streaming by increasing the fluid velocity, resulting in enhancing the conversion performance. G.H. Tang et al. [27] experimentally studied the flow characteristics of deionized water and PAM solution over a wide range of Re in a fused-silica-based circular microfluidics channel with specific geometrical features.

The combination of fluid rheology with different multiphysics fields provides a rich area for investigation. For instance, acoustic fields have been extensively studied in microfluidics systems for tasks such as mixing, particle manipulation, cell separation, and droplet control. Acoustic waves can induce localized pressure gradients and microstreaming flow patterns that strongly influence the rheological properties of non-Newtonian fluids [28]. In the same way, electric fields are also extensively utilized in microfluidics systems for various processes such as electroosmotic pumping, separation based on the dielectrophoretic principle, and electrowetting, etc. In addition, the presence of non-Newtonian polymer solutions with changing viscosity and elasticity introduces an additional layer of complications to perform such processes because of non-linear fluid behavior across the channel length [29]. Moreover, magnetic fields also play a crucial role in microfluidics systems, especially when dealing with magnetically responsive particles like ferroparticles that mainly depend on magnetic labelling [30]. In all these scenarios, it is crucial to study the interaction between fluid rheology with different external fields to design efficient and reliable microfluidics systems. Moreover, numerical simulations provide a controlled and systematic exploration of study parameters such as channel design, flow rate, fluid characteristics, and external fields. Furthermore, among all different available computational tools, COMSOL Multiphysics has become highly popular because of its ability to combine multiple physics fields in a single simulation framework [31]. This is crucial in microfluidics, where the flowing fluid is frequently influenced not only by pressure-driven forces but also affected by external fields such as acoustic, electric, and magnetic fields. Thus, through the integration of these multiphysics effects, a numerical simulation approach may provide deep insights into the performance and optimization of microfluidics systems while working with complex fluids. To-Lin Chen et al. [32] conducted an experimental investigation of the impact of fluid shear-thinning on the development of electrokinetic waves by adding the small amount of xanthan gum (XG) polymer to both high- and low-concentration Newtonian buffer solutions. The study revealed how shear-thinning behavior in non-Newtonian fluids affects electrokinetic instabilities in microfluidics channels. Kailiang Zhang et al. [33] proposed an efficient methodology to produce Newtonian and non-Newtonian fluids at chosen concentration using AC electrothermal flow. They found that fluid concentration control is directly impacted by the fluid properties and controlled by external means such as fluid velocity and voltage. A. Magesh et al. [34] examined the effect of an induced magnetic field on Jeffrey fluid using a peristalsis curved channel. It has been observed from the existing literature that most of the studies have mainly focused on investigating the rheological behavior of non-Newtonian fluids with single physics without considering the comparative study of three different physics such as acoustic, electric, and magnetic fields. This restrains the insufficient and frequently inaccurate prediction of rheological characteristics and interaction with external fields in microfluidics channels where modified rheological behavior and active field interactions are essential for controlling the shear rate and fluid dynamics. In addition, the existing studies also focused on simple microchannels geometries such as rectangular-shape, cylindrical-shape, T-shape, curved-shape, and expansion–contraction channels but without integration of complex geometrical features such as micropillar structures, etc. This may lead to further complication in predicting accurate rheological characteristics and fluid dynamics within the microchannel. In addition, many authors have also used theoretical and experimental approaches to study fluid rheology in microchannels without considering a numerical approach. These limitations in existing studies highlight the critical need for a more comprehensive investigation into the rheological characteristics of non-Newtonian fluids under the influence of three distinct external active fields. However, it has been thoroughly observed that there is still a significant gap in the study of fluid rheology of fluids with non-Newtonian characteristics in T-shape microfluidics microchannels integrated with complex micropillar structures. The novelty of this research work lies in conducting detailed multiphysics numerical investigations using COMSOL, encompassing three key physics such as acoustic fields, electric fields, and magnetic fields. This approach enables a deeper understanding of fluid rheological interactions with complex channel geometries and external active fields and their influence on the overall performance of microfluidics systems.

The main objective of this work is to numerically evaluate the fluid rheology of three different polymer solutions such as polyacrylamide (PAM), polyethylene oxide (PEO), and xanthan gum with non-Newtonian characteristics in a T-shaped microfluidics channel integrated with micropillar structures under acoustic, electric, and magnetic fields. For this purpose, COMSOL Multiphysics with laminar flow, pressure acoustic, electric current, and magnetic field physics is used with the Carreau–Yasuda fluid model to characterize the shear-thinning behavior of the fluids. The numerical simulations are performed with various input parameters such as Reynolds numbers (0.1, 1, 10, and 50), acoustic pressure (5 Mpa, 6.5 Mpa, and 8 Mpa), electric voltage (200 V, 250 V, and 300 V), and magnetic flux (0.5 T, 0.7 T, and 0.9 T). Moreover, the influence of these parameters on flow instabilities and unusual flow patterns is also examined inside a microchannel integrated with complex micropillar structures. The findings offer a valuable insight into improving channel design, performance, and functionality when dealing with complex fluids with non-Newtonian characteristics. Moreover, this study offers a deep understanding of fluid rheology under different active fields and helps bridge the gap between theoretical studies and experimental applications.

## 2. Research Methodology

### 2.1. Device Configurations

**Figure 1:**
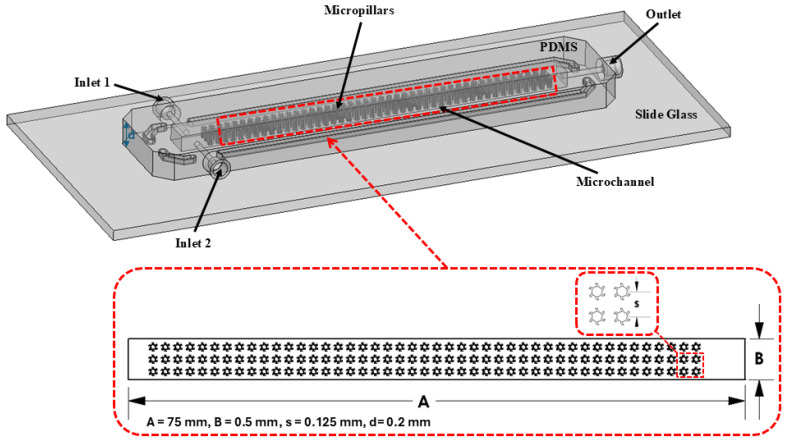
Schematic diagram of T-shaped microfluidics channel integrated with micropillar structures.

Figure 1 illustrates the schematic diagram of a T-shaped microfluidics channel integrated with complex micropillar structures (blade-shaped) with detailed geometrical configurations. The T-shape microchannel offers better control over fluid dynamics, is sensitive to external forces, produces localized gradients, and permits a complex interaction between external active fields and fluids. In addition, the T-shaped channel also helps in understanding the flow stability and ability to manipulate velocity profiles, etc. The micropillar structures are arranged and placed at repeated intervals that produce micro-vortices around the pillar structures and complex flow patterns by inducing local velocity gradients and recirculation zones. The integration of micropillar structures promotes efficient fluid mixing through chaotic advection and disruption of the laminar flow profile, thereby improving mass transfer, which makes it particularly effective for fluids with non-Newtonian characteristics. In addition, the proposed configuration also offers a controlled environment for investigating the influence of fluid rheology under varying shear conditions, especially for non-Newtonian fluids. The channel design contains two inlets which are perpendicular to the channel axis where the fluids with non-Newtonian characteristics are injected using a syringe pump and one outlet for collecting the mixed solution. The same fluid is used at both inlets but with different dye colors to make them distinguishable for observation. Moreover, the microfluidics device also consists of straight rectangular channel integrated with complex micropillar structures having a total length of 75 mm and width of 0.5 mm, and the spacing between the micropillars is 0.125 mm.

### 2.2. Material

Three sorts of polymer solutions with different rheological characteristics, such as 2000 ppm xanthan gum (XG), 1000 ppm polyethylene oxide (PEO), and 1500 ppm polyacrylamide (PAM), are used for numerical simulations [35]. The Carreau–Yasuda fluid model is employed to describe the non-Newtonian behavior of the fluids with a power index of 0.5, exhibiting the shear-thinning characteristics commonly observed in polymer solutions. The mathematical equation of the Carreau–Yasuda fluid model describes how the viscosity of a fluid varies with the applied shear rate and can be expressed as follows [36,37].

(1) $$\eta (\overset{•}{\gamma })=\eta_{\infty }+(\eta_{0}-\eta_{\infty }){\left[1+{\left(\lambda \overset{•}{\gamma }\right)}^{a}\right]}^{\frac{n-1}{a}}$$

In the above equation, $\eta (\overset{•}{\gamma })$ is the apparent viscosity, $\eta_{\infty }$ is known as the infinite shear viscosity of the fluid, $\eta_{0}$ is described as the zero-shear viscosity, λ is the time constant that is also known as control parameters between the Newtonian and power-law region, a is defined as Yasuda parameters, and *n* is the power index that demonstrates the shear-thinning behavior of the fluid. Table 1 lists the rheological properties of the polymer solutions used for the numerical simulations [35,38].

### 2.3. Fluid Domain and Meshing

**Figure 2:**
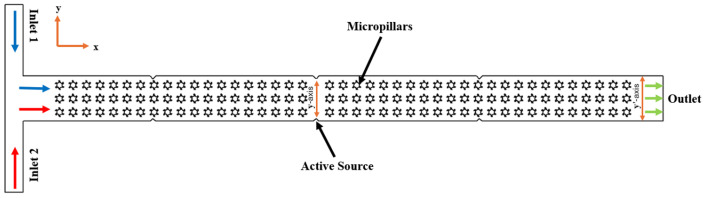
Computational domain for numerical simulations.

**Figure 3:**
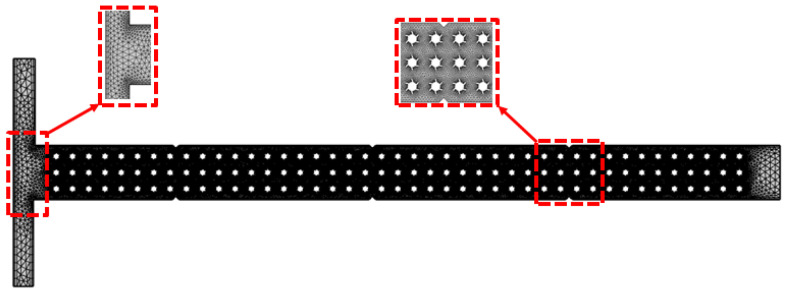
Meshing of T-shaped microfluidics channel integrated with complex micropillar structures.

Figure 2 illustrates the two-dimensional (2D) fluid domain of the T-shaped microchannel integrated with complex micropillar structures, which are arranged in a regular pattern along the channel length in order to induce secondary flow and stretching of the fluid interface, resulting in varying fluid rheology. The microchannel comprises two inlets where the two identical fluids are injected by using a syringe pump at different Reynolds numbers (Re) and one outlet for collecting the waste solution. The flowing fluids coming from the two different inlets are merged and flow through the micropillar region, where external active fields such as acoustic, electric, and magnetic fields are also employed. The flowing fluid within the microchannel is assumed to be laminar and incompressible and governed by the equation of continuity and the Navier–Stokes equation with non-Newtonian characteristics. In addition, meshing of the microchannel is created with 2D triangular elements, with the total number of elements and nodes being 83,646 and 52,250, respectively. The meshing of the microchannel is illustrated in Figure 3.

### 2.4. Mathematical Formulation

#### 2.4.1. Laminar Flow

Laminar flow physics generally demonstrates the dynamics of fluids under laminar flow conditions. In this study, the laminar flow of non-Newtonian polymer solutions is depicted using the incompressible continuity and momentum equations, where the viscous stress mainly relies on the shear-dependent viscosity. The Carreau–Yasuda fluid model generally demonstrates the shear-thinning behavior of the fluid by considering the viscosity and the local shear rate. This approach offers a consistent way to predict the velocity, pressure, and energy loss in a flowing fluid exhibiting non-Newtonian viscosity. The governing equations related to mass and momentum conservation, including the continuity and Navier–Stokes equations for incompressible non-Newtonian fluids, are explained below.

(2) ∇.u=0

(3) $$\rho \left(\frac{\partial u}{\partial t}+u.\nabla u\right)=-\nabla p+\nabla .[\eta (\overset{•}{\gamma })(\nabla u+{\left(\nabla u\right)}^{T})]+\rho g+f$$

where *u* is the fluid velocity, p is the pressure, ρ is the density, η is the dynamic viscosity of the fluid, $\eta_{0},\;\eta_{\infty }$ are the zero- and infinite-shear viscosities, respectively, λ is the time constant, *g* is defined as gravity, and *f* is the other body forces.

#### 2.4.2. Pressure Acoustics

Pressure acoustics are mainly based on the linearized acoustic wave equation, which assumes tiny harmonic pressure perturbations with varying base flow. In addition, this model describes the first-order linear acoustic fields while the fluid is flowing inside the microchannel. These equations also demonstrate how the acoustic pressure field deviates spatially under external excitation at a certain angular frequency. This computational approach also enables effective pressure distribution and its coupling with complex micropillar structures and other physical forces, such as hydrodynamic forces, etc. The governing equations corresponding to pressure acoustics with the frequency domain are given below [32,39].

(4) $$\nabla .\left(-\frac{1}{\rho_{c}}(\nabla_{p_{t}}-q_{d})\right)-\frac{k_{eq}^{2}}{\rho_{c}}p_{t}=Q_{m}$$

where $\rho_{c}$ is the acoustic density, $p_{t}$ describes the acoustic pressure, $q_{d}$ defines the moment or diploe density of the fluid, $k_{eq}$ is known as the effective wave number for the flowing fluid, and $Q_{m}$ represents the mono or mass source term. In addition, acoustic pressure can be split into scattered and background acoustic pressure fields, as given in the following Equation (5).

(5) $$p_{t}=p_{2}+p_{b}$$

where $p_{2}$ is the scattered acoustic pressure field and $p_{b}$ is known as the background acoustic pressure field. Moreover, the effective acoustic wavenumber demonstrates the relationship between the total acoustic wavenumber and its subcomponents inside the fluid medium. The mathematical relation is given below (Equation (6)).

(6) $$k_{eq}^{2}={\left(\frac{\omega }{c_{c}}\right)}^{2}-k_{z}^{2}$$

where *c* is the speed of sound, ω is the angular frequency, and $k_{z}$ is known as wavenumber component that describe the acoustic field out of the plane. Similarly, the following mathematical relation (Equation (7)) describes the linear relationship between acoustic pressure and particle velocity.

(7) $$v_{1}=-\frac{1}{i\omega \rho_{c}}\nabla p_{t}$$

where $v_{1}$ describes the first-order acoustic particle flow velocity, and $\nabla p_{t}$ is known as the gradient acoustic pressure showing the direction and rate of change in pressure in the space.

#### 2.4.3. Electric Current

The influence of an electric field on the fluid rheology within the microchannel integrated with complex micropillar structures is crucial to characterize the shear-thinning behavior of the flowing fluid. In this study, the electric field is computed using electro-quasistatic electric current-related governing equations. For this purpose, electric current physics with a frequency domain is used for numerical simulations using COMSOL Multiphysics. The formulation accounts for both conduction and displacement currents. The flowing fluid is assumed to be a homogeneous, isotropic, and electrically sensitive medium, where the local rheological characteristics affect only the hydrodynamic field. The governing equations related to electric current continuity and electric potential are given below (Equations (8)–(10)) [32].

(8) $$\nabla .J=Q_{j,v}$$

(9) $$J=\sigma E+j\omega D+J_{e}$$

(10) E=−∇V, D=εE

where *V* is the electric potential, $Q_{j,v}$ represents the volumetric current source, *E* is known as the field, *D* demonstrates the electric displacement, σ is described as the electrical conductivity of the flowing fluid, jωD represents the displacement current, and $J_{e}$ is known as current density.

#### 2.4.4. Magnetic Field

In order to describe the magnetic field distribution and its interaction with the flowing shear-thinning fluid inside the microchannel, magnetic field physics is used for numerical simulations. In this model, the time-varying displacement current is assumed to be neglected because the operating frequency is sufficiently minimal compared to the electromagnetic wave propagation frequency. The magnetic field model generally solves the magnetic vector potential, from where the magnetic flux density and field intensity of flowing fluid are drawn. Similar to the electric field, the magnetic field governed by the Carreau–Yasuda fluid model mainly does not directly influence the fluid rheology but affects the hydrodynamic behavior of the fluid. The magnetic-field-related governing equations depend on the magnetic material properties such as permeability and conductivity. The mathematical formulation related to the magnetic field with the frequency domain are expressed as follows [40,41].

(11) $$\nabla .H=J+J_{e}$$

(12) $$\nabla .B=\mu_{0}J_{tot}$$

(13) $$\nabla .\left(\frac{1}{\mu }\nabla \times A\right)=J_{e}+\sigma \left(-\frac{\partial A}{\partial t}-\nabla V+u_{0}\times (\nabla \times A)\right)$$

where *A* represents the magnetic potential, *B* is known as the magnetic flux, *H* represents the magnetic intensity, *J* is the induced current density, and μ defines the magnetic permeability.

### 2.5. Numerical Settings and Boundary Conditions

In this study, numerical investigation of a non-Newtonian Carreau–Yasuda fluid was carried out to examine its flow behavior and fluid rheology in a T-shaped microchannel integrated with complex micropillar structures considering the effects of acoustic, electric, and magnetic fields. For this purpose, COMSOL Multiphysics version 5.6 with four different physics, such as laminar flow, pressure acoustic, electric current, and magnetic field, is used to examine their influence on fluid motion and rheological behavior. A 2D steady-state laminar flow model was developed, incorporating the continuity and momentum governing equations, coupled with a constitutive relation with the Carreau–Yasuda fluid model to accurately represent the shear-thinning nature of the fluid. The governing equations for the incompressible, laminar flow of the non-Newtonian Carreau–Yasuda fluid model were solved using the Finite Element Method (FEA). In addition, Pardiso Direct Solver is used for numerical simulations, which offers high accuracy for complex and non-linear systems. Moreover, the convergence criterion was set to 10^−4^, with a maximum of 1000 iterations per solution. The computational domain was discretized with a non-uniform triangular mesh element with local refinement near the complex micropillar structures and T-junction to precisely resolve the velocity gradients and local shear effects. In addition, a relative convergence tolerance of 10^−6^ was employed for all dependent variables to ensure the accuracy of the solution.

Moreover, identical non-Newtonian fluids were introduced simultaneously at both inlets with equal flow rates, corresponding to Reynolds numbers (Re) of 0.1, 1, 10, and 50. The corresponding Reynolds numbers were determined based on the hydraulic diameter and average inlet flow velocities. Zero-gauge pressure boundary conditions were imposed at the outlet of the microchannel, while the channel and micropillar walls were considered as no-slip boundaries. Furthermore, external active fields were also coupled with the flow field to evaluate their impact on fluid motion and fluid rheology. For this purpose, three independent external fields, such as acoustic, electric, and magnetic fields, were coupled with the fluid domain. An acoustic field was established as a standing acoustic wave by applying an acoustic pressure of equal magnitude on both the upper and lower walls of the microchannel with opposite directions. Three different acoustic pressures of 5 Mpa, 6.5 Mpa, and 8 Mpa were applied with an excitation frequency of 500 kHz in order to evaluate the influence of acoustic intensity on fluid behavior as well as fluid rheology. Similarly, an electric field was also implemented as a uniform electric potential of 200 V, 250 V, and 300 V between the sidewalls of the microchannel, producing an electrokinetic body force on the charged fluid to examine the impact of the electric field on fluid motion and fluid rheology, particularly for a non-Newtonian fluid. In addition, uniform magnetic flux densities of 0.5 T, 0.7 T, and 0.9 T were imposed perpendicular to the channel axis in order to generate magnetohydrodynamic influences through the Lorentz forces to evaluate the impact of the magnetic field on flow behavior as well as fluid rheology. These multiphysics couplings were fully integrated with the fluid momentum equations in order to examine the interaction between the external fields and the flowing fluid.

### 2.6. Mesh Sensitivity Analysis

**Figure 4:**
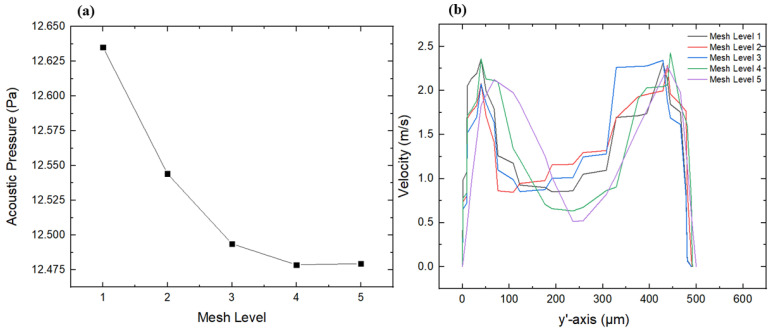
Mesh sensitivity analysis; (**a**) Variation in acoustic pressure for different mesh refinement levels, (**b**) Variation in velocity magnitude for different mesh refinement levels.

Mesh sensitivity plays an important role in performing FEA analysis in order to ensure an accurate solution. The accuracy of the computed outcomes as well as the computational cost substantially rely on the total number of elements and nodes within the mesh. In addition, mesh sensitivity confirms that further refinement does not importantly influence the computed results, confirming mesh independence. In this study, mesh sensitivity analysis was performed at different refinement levels until independence of the computed solution was reached. Comprehensive mesh sensitivity details are presented in Table 2. To study the mesh sensitivity, the maximum acoustic pressure and the outlet velocity profile at the outlet section (y’-axis) were computed for five different mesh refinement levels, as illustrated in Figure 4a,b. It is evident from both the tabular details and graphical representations that the variations in the computed parameters became negligible beyond a mesh refinement level of 4, with a relative error of less than 1%. Thus, a mesh refinement level of 4 was selected as the best mesh configuration, with a total number of elements and nodes of 83,666 and 52,256, respectively. This optimal configuration offers an appropriate balance between computational accuracy and cost.

## 3. Results and Discussions

### 3.1. Flow Field

**Figure 5:**
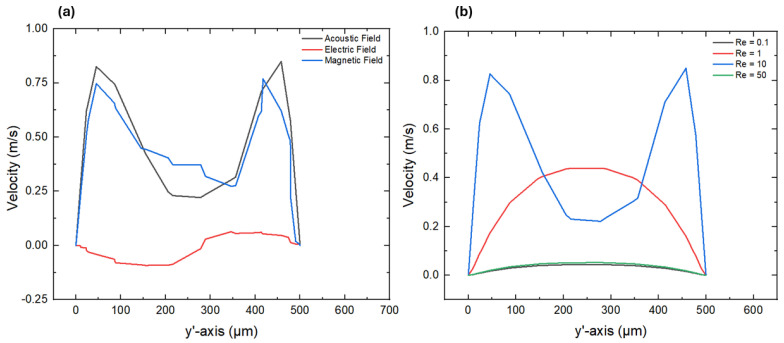
Velocity profiles at the outlet (y’-axis) (**a**) with different active fields and (**b**) with different Reynolds numbers.

Flow field analysis was performed to understand the fluid rheology of non-Newtonian fluids within the microfluidics channel, as it revealed the velocity magnitude and flow directions across the different cross-sections. This helped to identify the high- and low-velocity regions as well as micro-vortices induced by active fields within the microchannel, particularly in the presence of complex micropillar structures under laminar flow conditions. Figure 5a,b illustrate the velocity profiles at the outlet section (y’-axis) under different active fields as well as different Reynolds numbers. These velocities profiles reveal the transition between viscous-dominated and inertial-dominated regimes within the microchannel. An apparent variation in the velocity magnitude and distribution under the three different active fields, such as acoustic, electric, and magnetic fields, was observed, as shown in Figure 5a. The acoustic field exhibited the highest velocity magnitude of 0.84 m/s, revealing pronounced fluid fluctuations that enhanced momentum transfer within the microfluidics channel in the presence of complex micropillar structures. On the other hand, the magnetic field generated a moderate velocity profile by exerting magnetic body forces acting on the fluid, which generated localized acceleration zones and contributed to secondary flow patterns. The maximum velocity magnitude of 0.84 m/s was observed under the acoustic field followed by the magnetic field, with 0.76 m/s, and the electric field, with 0.06 m/s. The electric field exhibited strong electrokinetic effects, which helped to lower the velocity magnitude, leading to weaker fluid motion compared to the acoustic and magnetic fields. The acoustic field generated weaker acoustic standing waves within the microchannel, resulting in a 14 times lower velocity magnitude compared to the electric field. In addition, the symmetric double-peak nature of the velocity profiles indicates higher flow velocities near the channel wall. This kind of behavior primarily arose due to the shear-thinning characteristics of the non-Newtonian behavior and development of micro-vortices around the micropillar structures. Similar velocity profile behavior for different solutions and fluid models can be examined in [41,42]. Moreover, Figure 5b illustrates the velocity profiles for different Reynolds numbers such as 0.1, 1, 10, and 50 at the outlet section (y’-axis). A double-peak structure in the velocity profile can be observed in Figure 5b, indicating higher flow velocities near the channel walls and reduced velocities at the central region at lower Re values such as 0.1 and 1. The maximum velocity magnitudes at Re 0.1 and were are 0.04 m/s and 0.43 m/s, respectively. This flow pattern is a feature of the shear-thinning behavior noted in non-Newtonian fluids, where dynamic viscosity reduces with the increase in the applied shear rate, advancing the flow near the boundaries. As the Re value increased to 10 and 50, the overall flow velocities reduced up to 0.05 m/s, and the velocity profile became more uniform compared to the lower Re values such as 0.1 and 1. This implies a transition toward a more viscous-dominated flow regime with reduced inertial effects. Similar velocity profile behaviors in a rectangular-shaped microchannel with non-Newtonian fluids can also be observed in [43].

**Figure 6:**
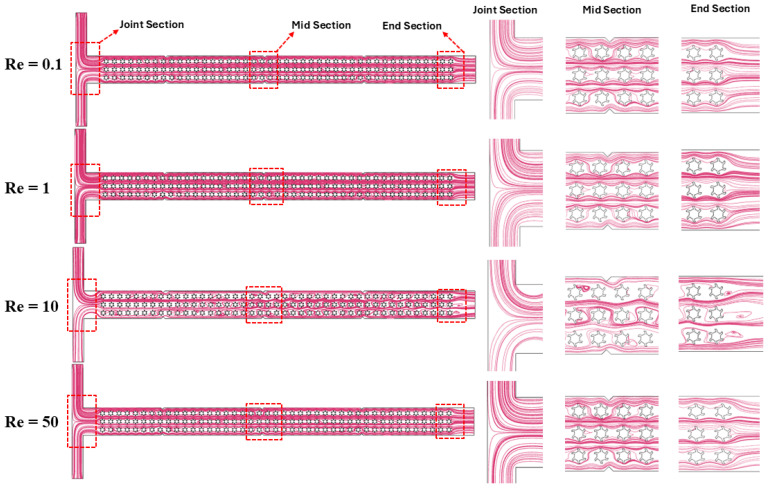
Velocity streamlines in a T-shaped microchannel at different Reynolds numbers.

Figure 6 describes the velocity streamline contours of the non-Newtonian fluids for different Re values such as 0.1, 1, 10, and 50 at various sections in the microfluidics channel. The variation in Re helps in understanding the flow regimes within the microchannel at different sections such as the T-joint, middle, and end. At Re 0.1, the flow is considered fully laminar, as indicated by smooth and parallel streamlines that specify stable and ordered fluid movement within the microchannel. As Re rises to 1 and then to 10, minor instabilities start to be observed, compared to Re 0.1, specifically in the joint section, where shear forces begin to induce secondary flow patterns. When Re increases to 50, the fluid flow behavior becomes more chaotic, particularly near the joint and end sections, where turbulence is more visible. An increasing distortion in the velocity streamlines and formation of micro-vortices can be clearly observed with the rise in Re. Overall, these streamline patterns indicate how the non-Newtonian polymer solution with its complex rheological characteristics moves from stable to more chaotic flow as Re increases. In addition, the molecular structure of the polymer solution also affects the flow patterns by changing the shear-thinning effects, resulting in flow instabilities. Similar studies are conducted in [9,44], showing how viscoelastic polymer solutions transition from laminar to chaotic flow at different Reynolds numbers.

### 3.2. Acoustic Field

**Figure 7:**
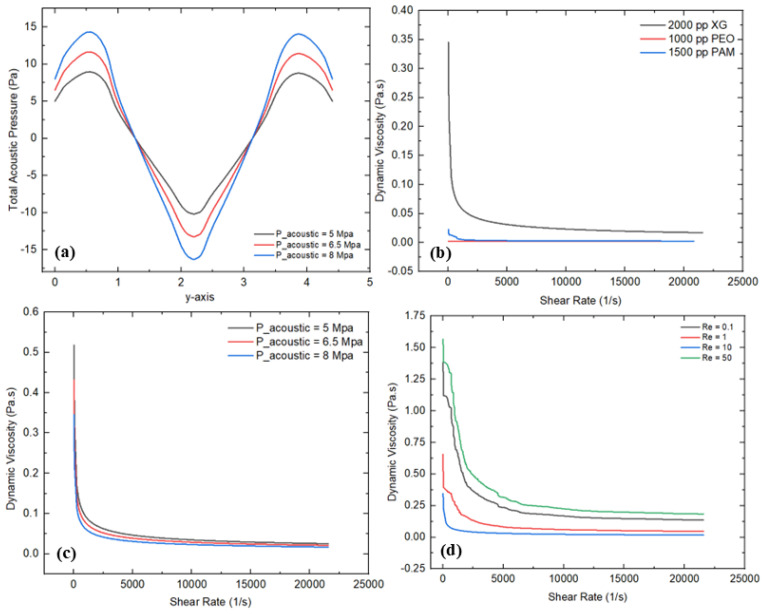
Rheological investigations for an acoustic field: (**a**) variation in total acoustic pressure at the center (*y*-axis) for different acoustic pressures, (**b**) variation in dynamic viscosity and shear rate for different polymer solutions, (**c**) variation in dynamic viscosity and shear rate for different acoustic pressures, (**d**) variation in dynamic viscosity and shear rate for different Re values.

This section describes the influence of acoustic fields on the fluid rheology of polymer solutions with non-Newtonian characteristics in a T-shaped microfluidics channel integrated with complex micropillar structures. Figure 7a–d represent the various graphical representations, showing the comprehensive rheological details. Figure 7a illustrates the variation in total acoustic pressure at the mid-section (*y*-axis) of the microfluidics channel under different acoustic pressures such as 5 Mpa, 6.5 Mpa, and 8 Mpa. The curves reveal sinusoidal fluctuations in total acoustic pressure at the central vertical axis, where the maximum and minimum acoustic pressure occurs near the acoustic source and at middle of the channel, respectively. In addition, the maximum value of the total acoustic pressure of 14.32 Pa is observed for an acoustic pressure of 8 Mpa, which is 1.6 times larger than the minimum value of the total acoustic pressure recorded at 5 Mpa. The transition of the total acoustic pressure from increasing to decreasing shows the significant influence of the acoustic field and micropillar structures on varying the acoustic standing waves. These acoustic standing waves induce secondary flow that produces a velocity gradient and disturbs the molecular structure of the fluid, while the complex micropillar structures generate localized turbulence and secondary flow, resulting in reducing the resistance to flow and influencing the rheological properties by varying the shear rate employed on the fluid within the channel. On the other hand, variations in the dynamic viscosity and shear were also investigated for different polymer solutions such as 2000 ppm XG, 1000 ppm PEO, and 1500 ppm PAM, as shown in Figure 7b. A strong shear-thinning behavior of the non-Newtonian fluids is observed, where the dynamic viscosity radically decreases with the increase in the applied shear rate. In addition, 2000 ppm XG shows a critical drop in dynamic viscosity, implying strong shear-thinning behavior. The main reason behind this variation in viscosity is due to their distinct molecular structures and interaction with shear forces. XG exhibits a flexible molecular structure that collapses and aligns the flow streamlines under shear rate, trigging strong shear-thinning behavior. Similarly, PEO and PAM represent moderate declines in viscosity because of their linear and branched chains, which are relatively less sensitive to shear, resulting in less reduction in viscosity, representing moderate shear-thinning behavior. It is examined that the dynamic viscosity is 17 times less in case of the PEO solution and 10 times in case of PAM compared to XG. Moreover, the variation in the dynamic viscosity and shear rate was also examined for different acoustic pressure values such as 5 Mpa, 6.5 Mpa, and 8 Mpa, indicating the significant influence of acoustic pressure on the shear-thinning behavior of the fluid within the microchannel, as illustrated in Figure 7c. It can be observed from the graphical representations that the dynamic viscosity decreases as the acoustic pressure increases. More acoustic pressure induces strong standing waves that cause stimulation of fluid molecules, resulting in high movement of fluid within the microchannel in the presence of complex micropillar structures. The maximum and minimum values of the dynamic viscosity of 0.51 Pa·s and 0.34 Pa·s are observed at 8 Mpa and 5 Mpa, respectively. A similar graphical trend can also be observed in [45], where they conduct rheological investigations based on PAM polymer solution with different concentrations, indicating the shear-thinning behavior of the fluid. Furthermore, the variation in the dynamic viscosity and shear rate was also investigated at different Reynolds numbers, such as 0.1, 1, 10, and 50, showing the significant influence of altering Re on the shear-thinning behavior of fluids, as shown in Figure 7d. As Re rises, the graphical trends show that the dynamic viscosity declines more quickly with the applied shear rate. This behavior indicates that at higher Re, the shear-thinning behavior of the fluid becomes more asserted in the presence of acoustic pressure as well as the complex micropillar structures.

**Figure 8:**
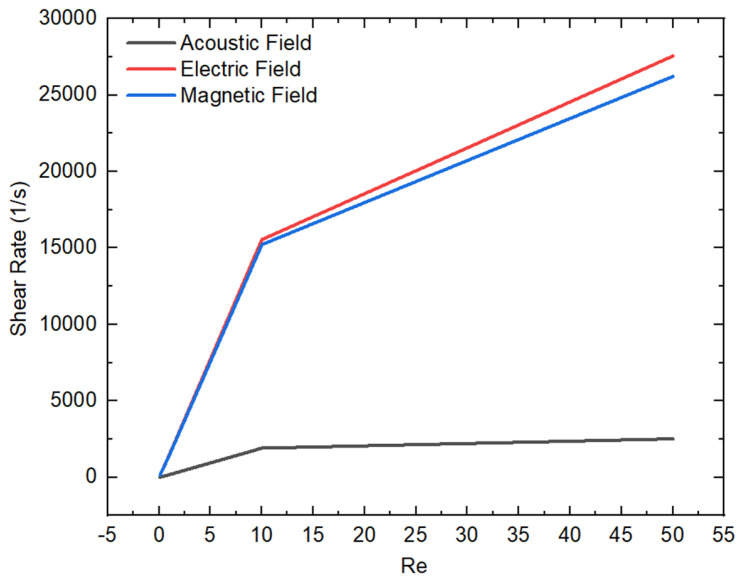
Variation in shear rate and Re at different external active fields.

Similarly, the relationship between the Reynolds number and shear rate is also illustrated for different active fields in Figure 8. This graph helps to understand the influence of varying the Reynolds number on fluid rheology by varying the shear rate across different active fields. As Re increases, the shear rate in the presence of the electric and magnetic fields rises noticeably, indicating that these fields accelerate the fluid flow within the microchannel. Alternatively, these stimulated flows significantly change the rheological characteristics of the fluid by increasing the fluid motion within the microchannel. Furthermore, both the electric and magnetic fields drastically increase the shear rate compared to the acoustic field. In the case of the electric field, the charged particles induce a direct force that aligns the fluid molecules along the flow streamlines, resulting in a strong driving force, leading to an enhanced fluid shear rate. Similarly, the magnetic field induces a Lorentz force implemented perpendicular to the flow direction, creating a resistance in the flow to some extent. On the other hand, the acoustic waves induce oscillating pressure waves, resulting in secondary flow that enhances the shear rate. Therefore, the shear rate is higher in the electric and magnetic field compared to the acoustic field.

**Figure 9:**
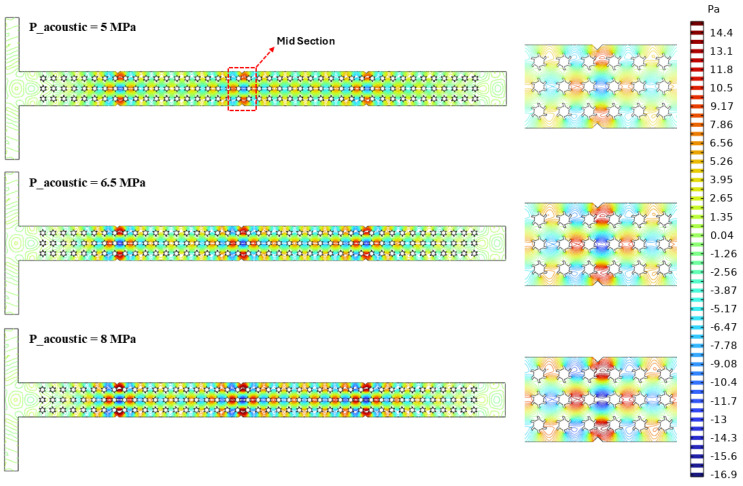
Acoustic streaming under different acoustic pressures.

Acoustic streaming is also presented in Figure 9 to investigate the influence of different acoustic pressures, such as 5 Mpa, 6.5 Mpa, and 8 Mpa, on the formation of micro-vortices within the microchannel in the presence of complex micropillar structures. It is evident from Figure 8 that as the acoustic pressure increases from 5 Mpa to 8 Mpa, the intensity and density of the pressure nodes and antinodes become more asserted in the case of an acoustic pressure of 8 Mpa, showing the generation of stronger acoustic standing waves within the microchannel. The uniform and symmetrical streamline patterns observed around the micropillar structures imply constructive interference, which improves the localized pressure gradients and strengthens the acoustic streaming influences within the microchannel. The elevated acoustic pressure observed at the acoustic pressure source leads to higher pressure fluctuations, resulting in stronger acoustic radiation forces that can vary the fluid motion and affect the polymer chain alignment within the microchannel. These fluctuations in the acoustic field directly influence the shear distribution and local fluid flow behavior, significantly affecting the fluid rheology within the microchannel in the presence of complex micropillar structures.

### 3.3. Electric Field

**Figure 10:**
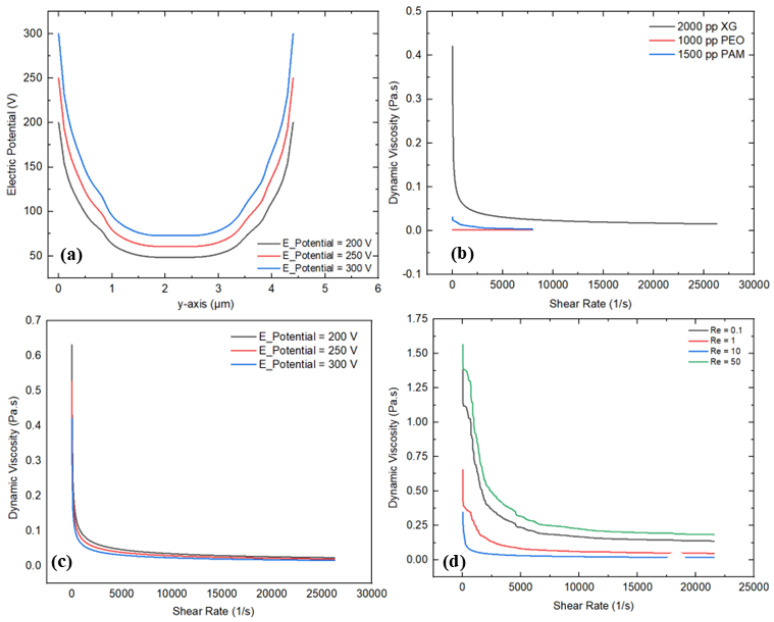
Rheological investigations for electric field: (**a**) variation in electric potential at the center (*y*-axis) at different applied voltages, (**b**) variation in dynamic viscosity and shear rate for different polymer solutions, (**c**) variation in dynamic viscosity and shear rate for different applied voltages, (**d**) variation in dynamic viscosity and shear rate for different Re.

An electric field significantly influences on the rheological behavior of non-Newtonian fluids, particularly within microfluidics channels. This section provides a comprehensive investigation into the impact of electric fields on the rheological behavior of polymer solutions exhibiting non-Newtonian characteristics in a T-shaped microfluidics channel integrated with complex micropillar structures. Figure 10a–d illustrate the various graphical descriptions, representing the detailed rheological information with varying input parameters. Figure 10a shows the variation in electric potential at the mid-section (*y*-axis) of the microchannel under different applied voltage values, such as 200 V, 250 V, and 300 V. It is evident that as the applied voltage enhances, the electrical potential rises across the vertical axis. The graphical trends show a parabolic shape, suggesting a non-uniform and precise distribution of electric potential along the axis. This trend reflects electrostatic interactions and the distribution of charged particles within the flowing fluid as the voltage rises. These charged particles alter the molecular structure of the flow streamlines and velocity profiles as well. These charged particles stimulate the fluid molecules and enhance the flow velocities and shear rate. In addition, the maximum electric potential is observed near the electric source, indicating strong electric field intensity in that region. In contrast, the minimum electric potential occurs at the center of the channel, suggesting weaker electric field intensity due to the presence of the complex micropillar structures. Alternatively, the variation in the dynamic viscosity and shear rate was also examined for different polymer solutions, such as 2000 ppm XG, 1000 ppm PEO, and 1500 ppm PAM, as shown in Figure 10b. Convincing shear-thinning behavior of the polymer solutions with non-Newtonian characteristics was observed, where the dynamic viscosity profoundly reduces with the increase in the applied shear rate. Additionally, a critical reduction in dynamic viscosity for the case of 2000 ppm XG was recorded, showing strong shear-thinning behavior compared to the other solutions, as their viscosities decreased at a moderate level. The main reason behind the strong shear-thinning behavior in the case of XG is due to its flexible molecular structure, which aligns the flow streamlines under the applied shear rate compared to the other polymer solutions (PEO and PAM). Moreover, the maximum value of the dynamic viscosity of 0.42 Pa·s was observed in the case of 2000 ppm XG, which is approximately 10 times greater than 1500 ppm PAM and 15 times greater than 1000 ppm PEO. Furthermore, the variation in the dynamic viscosity and shear rate was investigated for different applied voltages, such as 200 V, 250 V, and 300 V, as shown in Figure 10c. It can be observed from the results that increasing the applied voltage significantly influences the fluid rheology within the microchannel. Increasing the applied voltage, indicating a strong electrokinetic effect, leads to stimulating the movement of fluid molecules within the microchannel in the presence of complex micropillar structures. Computed values of the dynamic viscosity of 0.63 Pa·s, 0.52 Pa·s, and 0.42 Pa·s are noted at 200 V, 250 V, and 300 V, respectively. Similar investigations can also be observed in [46,47], where they studied how the dynamic viscosities of polymer solutions decrease with the applied shear rate under electric field effects, representing a strong influence of an electric field on fluid rheology. Also, the influence of the variation in the Reynolds number (0.1, 1, 10, and 50) on the fluid rheology is also investigated, as shown in Figure 10d.

**Figure 11:**
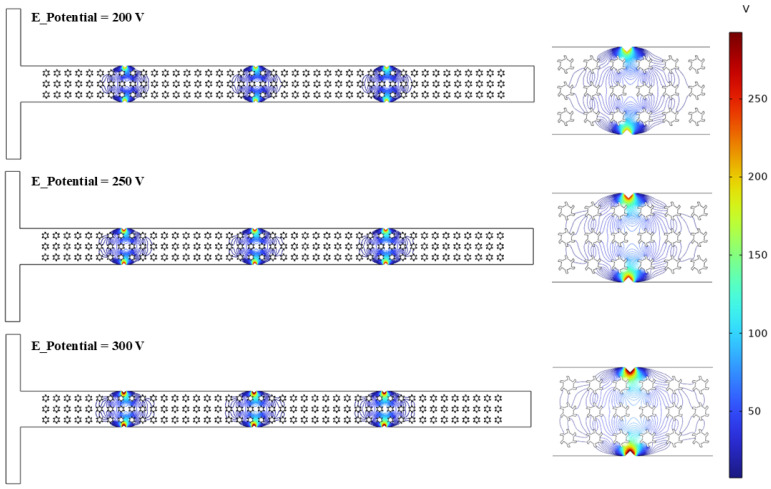
Electric potential streamlines under different applied voltages.

As Re increases, the dynamic viscosity trends rapidly decrease with the applied shear rate. This graphical behavior represents profound shear-thinning behavior in the presence of complex micropillar structures under an electric field. The maximum and minimum values of the dynamic viscosity of 1.56 Pa·s and 0.34 Pa·s were observed at Re values of 50 and 10, respectively. The main reason behind this variation is due to the non-Newtonian behavior of the polymer solutions. As Re increases from 0.1 to 10, the flow is considered as laminar, characterized by smooth fluid motion dominated by viscous forces. However, when Re increases beyond 10, the polymeric chains in the polymer solution begin to disentangle, resulting in an increment in shear resistance, leading to enhanced viscosity. Similar behavior can be observed in [48], where the viscosity is increased with the applied shear rate because of chain stretching in a contraction-type microchannel flow. Conversely, Figure 11 shows the electric potential streamlines for different applied voltages, such as 200 V, 250 V, and 300 V, in order to examine the influence of an electric field on the fluid motion and generation of micro-vortices within the microchannel in the presence of complex micropillar structures. It can be observed that as the applied voltage increases from 200 V to 300 V, the electric field intensity increases near the electric source, indicating more pronounced effects in that region. The more intense electric potential at higher applied voltage indicates a strong driving force, which influences the electrohydrodynamic behavior of the fluid within the microchannel. This electrohydrodynamic behavior significantly affects the fluid rheology of the polymer solution exhibiting non-Newtonian characteristics.

### 3.4. Magnetic Field

**Figure 12:**
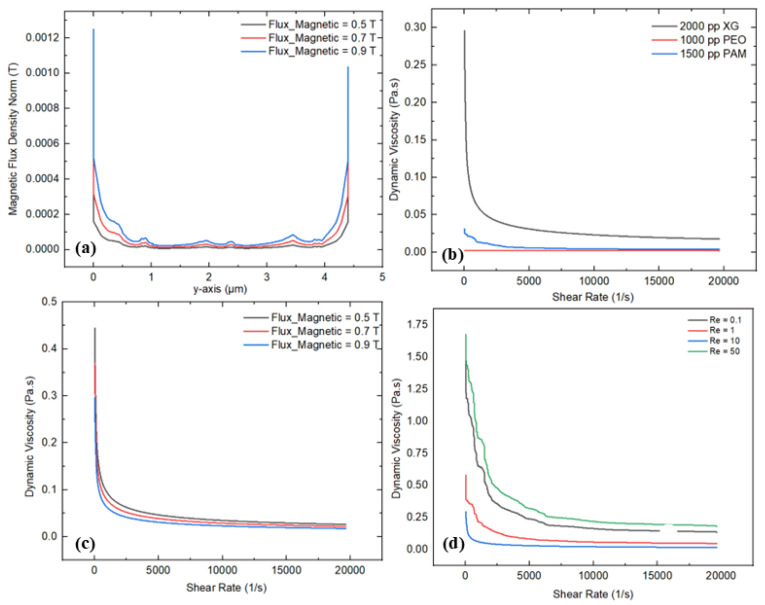
Rheological investigation for magnetic field: (**a**) variation in magnetic flux density at the center (*y*-axis), (**b**) variation in dynamic viscosity and shear rate for different polymer solutions, (**c**) variation in dynamic viscosity and shear rate for different magnetic flux values, (**d**) variation in dynamic viscosity and shear rate for different Re.

This section mainly discusses the effect of a magnetic field on the fluid rheology of non-Newtonian fluids within the microchannel. Figure 12a–d demonstrate the detailed rheological characteristics with varying input parameters. Figure 12a describes the magnetic flux density variation at the mid-section (*y*-axis) under different magnetic flux values, such as 0.5 T, 0.7 T, and 0.9 T. The graphical trends indicate strong magnetic fields near the source and a minimum at the center of the channel due to the presence of the micropillar structures. In addition, the flattened behavior in the graphical trends represents a weaker magnetic field due to the presence of complex-shaped micropillar structures. The maximum and minimum magnetic flux densities of 0.0012 T and 0.00038 T are observed at a magnetic flux of 0.9 T and 0.5, respectively. The main reason behind this decrement in flux density is due to their lowering the Lorentz forces in the case of a lower magnetic field that influence the fluid motion and streamlines within the microchannel. Conversely, the variation in the viscosity and shear rate for the different polymer solutions was also investigated, as shown in Figure 12b. Substantial shear-thinning behavior is noted in the polymer solutions with non-Newtonian characteristics, where the dynamic viscosity significantly decreases as the shear rate increases. In particular, the 2000 ppm XG solution shows a sharp reduction in viscosity, demonstrating stronger shear-thinning compared to the other solutions, whose viscosities drop at a slower rate. XG demonstrates a flexible molecular structure, which helps to align the fluid streamlines compared to the other polymer solutions, such as PEO and PAM. The dynamic viscosity values for the 2000 ppm XG, 1000 ppm PEO, and 1500 ppm PAM solutions are recorded as 0.29 Pa·s, 0.0023 Pa·s, and 0.03 Pa·s, respectively. Furthermore, the magnetic flux shows a significant deviation in dynamic viscosity and shear rate, indicating that increasing the magnetic flux strongly influences the fluid rheology within the microchannel, as shown in Figure 12c. Increasing the magnetic flux, representing a profound magnetohydrodynamic impact in the fluid, leads to stimulating fluid movement within the microchannel in the presence of complex micropillar structures. The maximum and minimum values of the viscosity are observed at 0.44 Pa·s and 0.29 Pa·s at a magnetic flux of 0.5 and 0.9, respectively. Moreover, the impact of various Re values on the fluid rheology was also investigated to observe the fluid behavior within the microchannel, as illustrated in Figure 12d. As Re rises, the dynamic viscosity decreases with the applied shear rate. This graphical behavior represents profound shear-thinning behavior in the presence of complex micropillar structures under magnetic fields. The maximum and minimum values of the dynamic viscosity of 1.67 Pa·s and 0.29 Pa·s were observed at Re values of 50 and 10, respectively.

**Figure 13:**
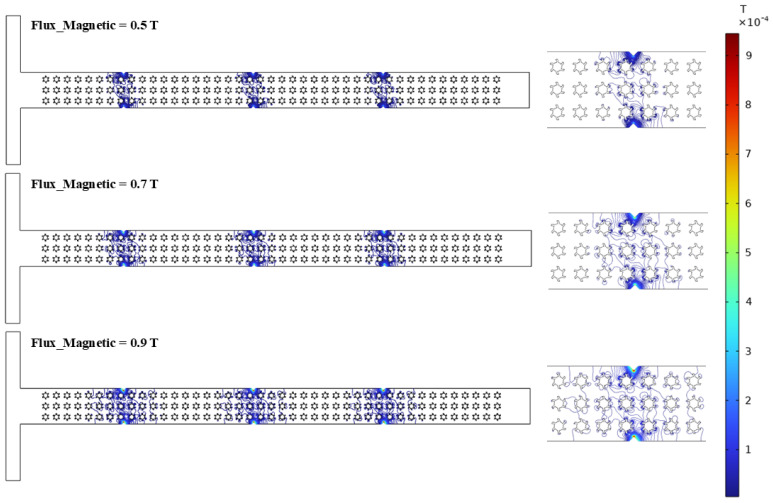
Magnetic field streaming under different magnetic flux values.

Figure 13 also represents the magnetic flux streaming, which helps investigate how different magnetic fluxes, such as 0.5 T, 0.7 T, and 0.9 T, influence the development of micro-vortices in the microchannel with complex micropillar structures. As the magnetic flux increases from 0.5 T to 0.9 T, the intensity of the magnetic field and flux density become more pronounced, indicating the generation of a stronger magnetic field distribution. As the magnetic flux rises from 0.5 T to 0.9 T, the magnetic field streamlines become more concentrated and exhibit a more complex streamline structure. At 0.5 T, the streamlines are relatively evenly distributed across the channel’s vertical axis, representing a weaker magnetic field interaction. However, the magnetic field streamlines are denser at a higher magnetic flux, suggesting that compelling magnetic forces lead to intensifying the fluid motion. Alternatively, these fluctuations directly influence the fluid rheology in the presence of complex micropillar structures.

## 4. Experimental Framework

**Figure 14:**
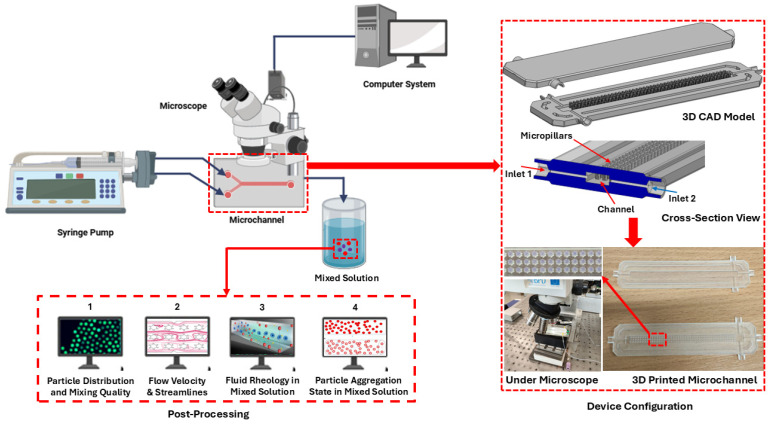
Proposed experimental framework.

Based on the findings, an experimental framework is proposed to study non-Newtonian fluid mixing in a T-shaped microfluidic channel integrated with complex micropillar structures under external active fields. The proposed experimental framework primarily consists of a syringe pump for injecting two different fluids containing microparticles at a controlled flow rate, a microchannel, an optical microscope, and a computer system for visualizing the fluid mixing and velocity streamlines inside the microchannel. The detailed framework is illustrated in Figure 14. Initially, the microfluidics channel will be printed using a high-resolution SLA printer (3D Creative, Vilnius, Lithuania) with a clear photopolymer resin material. Complex micropillar structures will be incorporated with regular patterns inside the microchannel to examine their impact on fluid mixing, particle stability, and the rheological behavior of the fluids. Three polymer solutions, such as 2000 ppm XG, 1000 ppm PEO, and 1500 ppm PAM, with non-Newtonian characteristics will be used for the experimental study. Additionally, a high-resolution microscope with camera will be connected to a computer system to visualize real-time flow streamlines and fluid mixing within the microchannel. Data acquisition will be used for storing data and image processing for comparative study with the numerical results. Finally, a mixed solution collected at the outlet will be used further for post-processing. Post-processing will comprise examining the particle distribution, mixing quality, rheological investigation, and particle aggregation. Overall, the proposed setup offers a comprehensive experimental framework for validating the numerical results and investigating complex fluids with non-Newtonian characteristics in a T-shaped microchannel under different external active fields.

## 5. Conclusions

This study numerically explored the fluid rheology of non-Newtonian fluids in a T-shaped microfluidics channel integrated with complex micropillar structures while incorporating external active fields (acoustic, electric, and magnetic). For this purpose, COMSOL Multiphysics with laminar flow, pressure acoustic, electric current, and magnetic field physics was used to investigate the fluid rheology within the microchannel. Three polymer solutions, such as 2000 ppm xanthan gum (XG), 1000 ppm polyethylene oxide (PEO), and 1500 ppm polyacrylamide (PAM), were used as non-Newtonian fluids with the Carreau–Yasuda fluid model to characterize the shear-thinning behavior. The findings emphasize the significant influence of active fields on fluid dynamics and rheological behavior. The key conclusions obtained from this study are summarized below.

1. The integration of external active fields (acoustic, electric, and magnetic) and the incorporation of complex micropillar structures significantly influences the rheological behavior and fluid dynamics of non-Newtonian fluids while flowing through the microchannel. The combination of these factors can significantly help to modify fluid behavior and rheological characteristics and provide better shear control in a microfluidics channels.
2. The flow field demonstrates that the external active fields and complex micropillar structures significantly influence the flow behavior and velocity profiles of non-Newtonian fluids within the microfluidics channel. The maximum velocity magnitude of 0.84 m/s is observed under an acoustic field, followed by a magnetic field, with 0.76 m/s, and an electric field, with 0.06 m/s. These findings reveal the substantial role of active fields and Re in the design and modification of microfluidics channels.
3. The acoustic field reveals that the acoustic pressure and complex micropillar structures significantly influence the fluid rheology. As the acoustic pressure rises from 5 Mpa to 8 Mpa, the total acoustic pressure increases by 1.6 times the minimum values at 5 Mpa. This may cause a reduction in dynamic viscosity from 0.51 Pa·s at 8 Mpa to 0.34 Pa·s at 5 Mpa. In addition, the rheological behavior is also influenced by different polymer solutions, as XG exhibiting 17 times reduction in viscosity compared to the other polymer solutions. The findings demonstrate that an acoustic field can be beneficial to stabilize laminar flow conditions and improve the rheological behavior where uniform flow is needed.
4. The electric field induces a higher shear rate compared to the other external active fields, resulting in more chaotic flow patterns within the microchannel and an evident reduction in dynamic viscosity, indicating convincing shear-thinning behavior. As the applied voltage increases from 200 V to 300 V, the dynamic viscosity reduces from 0.63 Pa·s to 0.42 Pa·s. In addition, XG also demonstrates substantial shear-thinning behavior, with a dynamic viscosity of 0.42 Pa·s, which is nearly 10 and 15 times higher than PAM and PEO, respectively. Moreover, higher voltages produce strong electric field intensities and micro-vortex formation, resulting in significant variation in velocity profiles at the outlet section (y’-axis) compared to the other external active fields. This electric field behavior can be leveraged to design and optimize microfluidics devices by modifying rheological properties and controlling shear rates.
5. Similarly, the magnetic field also generates moderate shear rate changes and made secondary flow patterns within the microchannel, indicating a clear shear-thinning behavior. The maximum magnetic flux densities recorded are 0.0012 T at 0.9 T and 0.00038 T at 0.5, with a subsequent reduction in dynamic viscosity from 0.44 Pa·s at 0.5 T to 0.29 Pa·s at 0.9 T. In addition, XG demonstrates significant shear-thinning behavior; as the shear rate increases, the dynamic viscosity decreases from 0.29 Pa·s to 0.0023 Pa·s. These findings can be beneficial to control the flow characteristics and for shear control for specified applications such as enhancing mixing with complex fluids.
6. Moreover, the influence of varying the Reynold number on the fluid rheology was also studied. As Re increases from 0.1 to 50, the flow transitions from laminar to more chaotic, resulting in a higher shear rate. In addition, more chaotic flow is observed under electric and magnetic fields, while less chaotic flow is examined in case of an acoustic field, which maintains a more uniform flow within the microchannel.
7. Based on the current findings, an experimental framework is proposed to study non-Newtonian fluid mixing in a T-shaped microfluidics channel under external active fields. An experimental framework comprises a syringe pump, optical microscope, microchannel, and computer system. The microchannel device is fabricated using a high-resolution SLA printer with clear photopolymer resin material. The post-processing will include examining the particle distribution, mixing quality, fluid rheology, and particle aggregation. Overall, the proposed setup offers a vigorous framework for validating numerical models and studying complex non-Newtonian fluids under different external active fields.

## Figures and Tables

**Table 1:** Rheological properties of polymer solutions used for numerical simulations.

Polymer	$\eta_{0}$ (mPa·s)	$\eta_{\infty }$ (mPa·s)	*n*	λ (ms)	EI
2000 ppm xanthan gum (XG)	1740	1.8	0.33	≈0	≈0
1000 ppm polyethylene oxide (PEO)	2.4	1.5	0.85	1.5	0.37
1500 ppm polyacrylamide (PAM)	1200	1.6	0.5	800	0.45

**Table 2:** Details of mesh sensitivity analysis.

Mesh Refinement Level	Number of Elements	Number of Nodes	Maximum Acoustic Pressure (Pa)	Relative Error (%)
1	50,193	31,678	12.6350	1.246
2	50,198	31,781	12.5440	0.517
3	79,300	49,981	12.4936	0.113
4	83,666	52,256	12.4785	0.008
5	130,283	76,157	12.4795	--

## Data Availability

The original contributions presented in the study are included in the article, further inquiries can be directed to the corresponding author.
